# N-of-1 trials in the clinical care of patients in developing countries: a systematic review

**DOI:** 10.1186/s13063-018-2596-5

**Published:** 2018-04-23

**Authors:** Chalachew Alemayehu, Jane Nikles, Geoffrey Mitchell

**Affiliations:** 1 Faculty of Medicine, University of Queensland, Brisbane, Australia; 2 UQCCR, Brisbane, Australia

**Keywords:** N-of-1 trials, Clinical trials, Developing countries, Systematic review

## Abstract

**Background:**

N-of-1 trials have a potential role in promoting patient-centered medicine in developing countries. However, there is limited academic literature regarding the use of N-of-1 trials in the clinical care of patients in resource-poor settings.

**Objective:**

To assess the extent of use, purpose and treatment outcome of N-of-1 trials in developing countries.

**Method:**

A systematic review of clinical N-of-1 trials was conducted between 1985 and September 2015 using PubMed, Embase, CINAHL, Web of Science and the Cochrane Central Register of Controlled Trials. Grey literature databases and clinical trial registers were also searched. This review included randomized, multi-cycle, crossover within individual patient trials involving drug intervention. Quality assessment and data extraction were conducted by two independent reviewers.

**Result:**

Out of 131 N-of-1 trials identified, only 6 (4.5%) were conducted in developing countries. The major reason that N-of-1 trials were used was to provide evidence on feasibility, effectiveness and safety of therapies. A total of 72 participants were involved in these trials. Five of the studies were conducted in China and all evaluated Chinese traditional medicine. The remaining study was conducted in Brazil. The completion rate was 93%. More than half, 46 (69%) of subjects made medication changes consistent with trial results after trial completion.

A number of threats to the validity of the included evidence limited the validity of the evidence. In particular, the estimated overall effect in four of the included studies could have been affected by the “carry over” of the previous treatment effect as no adequate pharmacokinetic evidence regarding traditional medicines was presented.

**Conclusion:**

The prevalence and scope of N-of-1 trials in developing countries is low. A coordinated effort among government, clinicians, researchers and sponsor organizations is needed to increase their uptake and quality in developing countries.

**Systematic review registration:**

PROSPERO CRD42015026841.

**Electronic supplementary material:**

The online version of this article (10.1186/s13063-018-2596-5) contains supplementary material, which is available to authorized users.

## Background

Many people take medications that will not help them [1–3]. This is because current medical care primarily relies on randomized controlled trials (RCTs), which, under the assumption of no heterogeneity, estimate a constant effect size or difference between control and intervention populations. By contrast, physicians in routine clinical practice deal with individual patients whose responses may differ markedly from the average. The US Food and Drug Administration (FDA) guideline on personalized medicine (PM) acknowledges that there are considerable numbers of non-responders to medications used for chronic diseases [4]. Moreover, drug toxicity can vary among racial and ethnic groups [5]. This challenge moved the world into a new perspective, whereby clinical practice developed increased appreciation of individual variation, creating the platform of patient-centered medicine (PCM) [6, 7]. There is also an increasing demand for objective evidence to make clinical decisions – the quest for solid criteria to claim that one intervention works better than the other.

PCM in developed countries has reached a level where individual genetic variations that contribute to disease can be identified and targeted for treatment. For example, in the USA, legislation to promote research and practices aimed at personalizing medicine [8] and guidelines to include pharmacogenomics biomarkers on drug labels [6] have been introduced. In 2010, 11% of the labels of the top 200 medications sold in the US included pharmacogenetic information, a 10-fold increase from the 2003 estimation [9]. The development of pharmacogenetic-based PCM has paramount importance for the developing world. However, for several reasons, patients in developing countries are far from being able to utilize advancements in genetic medicine. According to the World Health Organization (WHO), use of costly initiatives like pharmacogenomics by countries able to afford this will widen the existing equity gap between developed and developing countries [10].

Moreover, there are other challenges such as traditional medicine use and use of untested generic drugs. Insufficient medicine regulation and enforcement in developing countries raises uncertainty about the quality of clinical care that physicians give to individual patients. Due to cost and resource constraints, the contribution of western-style pharmacogenetic medicine to address the lack of evidence will be low.

According to the World Bank, most developing countries have a low Gross National Income (GNI) per capita – under US$4036 [11]. They have a disproportionately high burden of non-communicable chronic disease (NCD) [12]. These countries often lack strong medicine regulation and enforcement rules [13]. There are times where this leads retail pharmacies and drug stores to welcome poor-quality generic drugs whose interchangeability against branded products is not well established [14, 15].

### N-of-1 trials

Where appropriate, another type of PCM, namely N-of-1 trials, offers an objective, efficient and cost-effective method of personalizing treatment and improving the quality of clinical care.

N-of-1 trials can provide a pragmatic clinical means of addressing individual variation in treatment response. N-of-1 trials are multi-cycle, double-blinded, controlled crossover trials conducted within individual patients [16–18]. They provide the strongest available evidence of treatment efficacy to inform decisions for the individual patient [19]. As a principle, N-of-1 trials require relatively stable symptoms or diseases, and test medications with short half-lives and rapid measurable responses [18, 20].

Chronic disease management using N-of-1 trials can improve patient management and save health costs [21, 22]. Thus far, N-of-1 trials have been used to address several challenges in clinical care; to determine optimal therapy for individual patients [23], to identify cost-effective treatment options [23] and to prove therapeutic equivalence of generic drugs [24, 25].

The pragmatic use of N-of-1 trials for assessing the comparative effectiveness of different therapeutic options and as a means of formally assessing the interchangeability of different brands of the same medicine is documented [26, 27]. A recent article reported a comprehensive review of three types of crossover designs, including N-of-1 randomized trials for addressing drug interchangeability [28].

To date, using the principle of N-of-1 trials, some developed countries have accumulated decades of experience in improving the quality of clinical care for individual patients. However, N-of-1 trials are not known in most developing countries. As the philosophy and practice of treatment optimization is less developed in developing countries, tailoring patient treatment is not often done proactively. When it is done, it comes at the expense of patient suffering and economic cost (See Table 1). Patients in resource-poor settings have the right to be provided the best possible available cost-effective treatment that works for them. By promoting individualized patient care, N-of-1 trials have the potential to improve the quality of clinical care given for individual patients in developing countries.

**Table 1:** Issues with the current process of assessing medicine effectiveness- factors that hamper appropriate medical care of patients in developing countries

Process/system factors: accessibility of health facilities, lack of updated treatment guidelines, cost of treatment.	
Physician factors: lack of knowledge on evidence-based medicine and research, misconceptions by physicians on patient’s treatment claims, no/low accountability of physicians for inappropriate treatment, lack of time.	
Patient factors: misconceptions of medicines and health conditions, low literacy level, low economic condition.	

What is not known is the extent to which N-of-1 trials are already employed in developing countries, and the uses to which they are put.

## Methods

### Overview

We conducted a systematic review of N-of-1 clinical trials published in journals indexed by PubMed, CINAHL, Web of Science and the Cochrane Central Register of Controlled Trials as well as publications from grey literature and unpublished sources from International Trial Registry Platforms between 1985 and 2015. The protocol for this review was developed based on the PRISMA Statement [29] and is registered at PROSPERO (PROSPERO CRD42015026841). The PRISMA Checklist can be found in Additional file 1. The review did not require Human Subjects Approval.

### Eligibility criteria

To be included in the review, a trial had to meet the following criteria; the trial had to:

1. Be conducted in humans
2. Be conducted in developing countries as defined by the World bank [11]
3. Involve randomization of treatments within blocks or pairs, crossover of interventions, individual patients or series of patients, and single patients as the unit of analysis
4. Evaluate pharmacological interventions (both modern and traditional medicine)
5. Report the purpose of the trial, number of patients involved, completion rate, number of subjects who responded to the test drug, and post-trial completion decision

### Information sources and searches

Studies published in English were considered for inclusion in this review. Besides, articles published in a language other than English were considered if they had published English language abstracts. Studies published between 1985 and September 2015 were included in this review. The search strategy covered both published and unpublished studies. A three-step search strategy was utilized for published articles. Firstly, an initial limited search of MEDLINE and EMBASE was undertaken, followed by analysis of the text words contained in the title and abstract, and of the index terms used to describe articles. A second search using all identified keywords and index terms was then undertaken across all included databases. Finally, the reference lists of all identified reports and articles were hand searched for additional studies. Unpublished studies were searched for at ClinicalTrials.gov and the WHO International Clinical Trials Registry Platform. The following grey literature databases were also searched: OAIster, Open Grey, National Library of Australia Trove and Proquest Digital Dissertations. Search terms included a range of terms describing potential N-of-1 trials in the title or abstract: N-of-1, single-case trial, single-subject research, single-case experimental design, single-patient study, single-patient trials, single-case trials, and single-patient trial.

### Assessment for inclusion and data collection

**Fig. 1:**
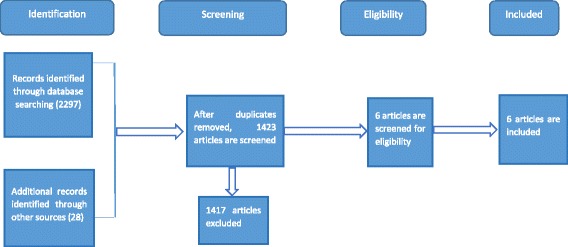
PRISMA flow chart for study selection

Titles and abstracts of all the retrieved bibliographic records were screened for potentially relevant articles. Full texts of potentially eligible records passing the title and abstract screening process were retrieved and examined according to the Cochrane Handbook Section 8.5.a for RCTs and section 16.4.3 for crossover trials [30]. A PRISMA flow chart outlines the study selection process [29] (Fig. 1).

Quantitative data were extracted from papers included in the review using an extraction tool adapted from the PRISMA Statement [29], and the CONSORT extension for reporting N-of-1 trials (CENT) [31] Checklist (Tables 2 and 4). We defined an N-of-1 as a trial that employed randomized treatment episodes to evaluate pharmacological interventions in a single patient.

**Table 2:** Characteristics of studies of N-of-1 tests in developing countries

1st author, country	Design	Rationale	Participants	Measures	Intervention	Outcomes
Huang, et al., China (2014) [32]	Randomized, double-blind, crossover, within individual patient	Lack of sufficient evidence on effectiveness of the therapy	1 man, 2 women, aged 18–75 years, diagnosed with stable bronchiectasis	Primary: patient self-rated symptom score for cough, expectoration, shortness of breath, chest pain and fatigue Secondary: 24-h sputum volume and drug safety	Herbal decoction vs control decoction	All three patients showed non-significant improvement from the test TCM. One patient preferred the herbal decoction over the standard one after trial completion
Yuhong, et al,, China, (2012) [33]	Randomized, double-blind, crossover, within individual patient	Lack of sufficient evidence on effectiveness of the therapy	15 men, 35 women, aged 25–65 years, with a clinical diagnosis of deficiency of kidney-Yin	Primary: individual completion rates, response rate, and post-N-of-1 RCT decision Secondary: self-rated symptom score on Likert scale and SF-36 questionnaire to measure perceived health and quality of life	*Liuwei Dihuang* decoction (LDD) vs placebo	Only 3 (6.38%) responded, 28 (59.57%) did not respond, and 16 (34.05%) were possible responders. 29 (66%) patients changed medication after the trial
Wang et al., China (2010) [35]	Randomized, double-blind, crossover, within individual patient	Lack of sufficient evidence on effectiveness of the therapy	6 men, 5 women, aged 45–66 years, with diagnosis of mild-moderate hypertension	Effectiveness: change in blood pressure (home and clinic measurements) Safety: respiratory rate, heart rate, routine blood test for liver and kidney function, urine test, routine ECG	High-dose vs low-dose *Bezoar* anti-hypertension capsule plus simulation placebo	Home BP measurements showed significant reduction only in SBP. Clinic BP measurements showed significant reduction both in SBP and DBP from the high-dose TCM (*P* < 0.001) There was no increased risk of adverse events from high-dose *Bezoar*
Yu et al,, China (2012) [36]	Randomized, crossover, within individual patient	Lack of sufficient evidence on effectiveness of the therapy	3 men, aged 52, 57 and 59 years with diagnosis of chronic kidney disease (CKD) of third stage	Individual patient main symptom score. Change in serum creatinine and creatinine clearance rate	Chinese medicinal decoctions plus the routine basic treatment vs only the routine basic treatment	Individual patients’ main symptom was significantly improved in the treatment phase (*P* < 0.01). Two patients showed improved serum creatinine and creatinine clearance rate
Zhang, et al., China (2012) [37]	Randomized, crossover, within individual patient	Lack of sufficient evidence on effectiveness of the therapy	4 patients, all male, ages 50, 61, 68 and 76 years, with diagnosis of hypertensive intracerebral haemorrhage	Patient main symptoms; IL-6, morphology index and clinical curative effect evaluation (the degree of encephaloedema and cerebral infarction)	Standard treatment plus TCM, acupuncture and moxibustion; Traditional Chinese manipulation vs standard treatment plus TCM	TCM symptom scores of all patients were significantly improved (*P* < 0.01). IL-6 of all patients was significantly reduced (*P* < 0.01). Scores of Morphology Index and Clinical Curative Effect were also improved from the treatment phase
Louly et al., Brazil (2009) [34]	Randomized, double-blind, crossover, within individual patient	Lack of optimal therapy to treat cough in patient	55-year-old female patient with dry cough secondary to interstitial pneumopathy	Primary outcome: the intensity of daytime and night-time cough measured by a visual analog scale and patient’s perception regarding her health state	Tramadol 50 mg compared with placebo	The patient’s condition as measured by visual analog scale significantly improved compared with the test drug (*P* < 0.001)

*BP* blood pressure, *DBP* diastolic blood presure, *RCT* randomized controlled trial, *SBP* systolic blood pressure, *TCM* Traditional Chinese Medicine

Included papers were reviewed by two independent reviewers (CA, JN) and records were compared between reviewers to ensure accuracy of data extraction. Any disagreements that arose between the reviewers were resolved through discussion. The data extracted included design, participants, measures, type of intervention, outcomes, number of planned treatment cycles, treatment length, washout, blinding, outcome measurement, responder definition, method of analysis, number of individuals completing the trial and number of post-N-of-1 RCT decisions which favor trial results.

### Data synthesis

The findings are presented in narrative form summarizing the data, which are presented in table form. Frequencies and percentages are reported. The goal of the review was to summarize the extent of N-of-1 use, the purpose for conducting them, outcomes and the subsequent treatment decisions after the trial. For this reason, we did not conduct a meta-analysis.

## Results

### Study selection

After removing the duplicates, the topic and abstracts of 1395 published and unpublished articles were reviewed to determine which were within the scope of this review. Figure 1 shows the study selection approach and the number of publications obtained. The initial assessment excluded 642 irrelevant publications.

Of the remaining 753 articles, the design and intervention of 131 articles met our definition for N-of-1 clinical studies. These articles were then subjected to review by the country in which they were conducted. One hundred and twenty-eight articles which were/are being conducted in developed countries were excluded. The remaining three articles were examined and included in the review [32–34]. To capture any additional N-of-1 trials, we hand searched references of excluded reviews and the three articles included the review. Twenty-eight additional articles were identified in this process. Out of the 28 articles identified, the abstracts of three articles [35–37] met our inclusion criteria. However, the full texts of these articles were published in Chinese journals in Mandarin. Thus, these three articles [35–37] were translated to English by a native Chinese speaker.

A total of six (five articles conducted in China [32, 33, 35–37] and one article from Brazil [34]) were included in this systematic review. Characteristics and synthesis of these included studies are displayed in Tables 2, 3 and 4, respectively.

**Table 3:** Quality of studies included

Author (date)	Sources of risk of bias							
	Selection bias (random sequence generation)	Selection bias (allocation concealment)	Reporting bias (incomplete outcome data)	Reporting bias (selective reporting)	Performance bias (blinding of participants and clinicians)	Inadequate cycles (risk of error – especially type 2)	Appropriateness of treatment for design	Appropriate washout period
Huang, et al., (2014) [32]	Low^a^	Low	Low	Low	Low	Low	Unclear	Low
Yuhong, et al.,(2012) [33]	Low	Low	Low	Low	Unclear	Low	Unclear	High
Wang et al., (2010) [35]	Low	Low	Low	Low	Low	Low	Unclear	High
Yu, et al., (2012) [36]	Low	Unclear	Low	Low	High	Low	Unclear	High
Zhang, et al., (2012) [37]	Low	Unclear	Low	Low	High	Low	Unclear	High
Louly, et al., (2009) [34]	Low	Low	Low	Low	Low	Low	Low	Low

^a^Level of risk

**Table 4:** Treatment characteristics of N-of-1 tests in developing countries

Variable	Number (%)
Type of medical intervention	
Traditional medicine	5 (83%)
Modern medicine	1 (17%)
Number of planned treatment cycles	
3 cycles	6 (100%)
Number of crossovers	
2	6 (100%)
Treatment length	
≥ 2 weeks	6 (100%)
Washout	
5–9 days	4 (67%)
2 days	2 (33%)
Number of trials blinded	4 (67%)
Outcome measurement (multiple answer)	
Patient self-rated symptom score	6 (100%)
Other measurement tools or questionnaires	5 (83%)
Responder definition	
P value < 0.05	4 (67%)
Visual analogue scale (not statistical) difference specified	2 (33%)
Clinical (not statistical) difference specified	2 (33%)
Method of analysis	
Pooled analysis (using methods other than Bayesian)	2 (33%)
Wilcoxon signed rank test/non-parametric	1 (17%)
Mean difference	2 (33%)
Paired *t* test	4 (67%)
Number of individuals who participated	72
Proportion (%) of individuals completing the trial (completion rate)	67 (93)
Proportion (%) of post-N-of-1 RCTs decisions which favor trial results	46 (69)

There were also three academic literature reviews on N-of-1 trials both in and outside the medical field [23, 38, 39]. The first review, published in 2010, was a systematic review of N-of-1 trials with and without pharmacological intervention [23]. In 2013, Duan et al. reviewed some of the academic literature to critically evaluate the need for further methodological developments [38]. It was not a full systematic review. Third, a systematic review which included N-of-1 articles with psychological and behavioral interventions was published recently [39] .

### Risk of bias within studies

A domain-level assessment of risk of bias was done to evaluate the following eight potential sources of bias for N-of-1 trials [30] (Table 3): random sequence generation, allocation concealment, incomplete outcome data, selective reporting, blinding of participants and personnel, number of treatment cycles, appropriateness of treatment for the design and adequacy of washout period.

The number and the scope of N-of-1 trials in developing countries is low. Of the 131 N-of-1 articles identified, only 6 (4.5%) were conducted in developing countries. Five of them were conducted in China to evaluate Traditional Chinese Medicine [32, 33, 35–37]. The sixth study [34], which evaluated modern medicine, was conducted in Brazil (Table 2). Seventy-two patients, with a range of 1–47 participants in each study, were involved in the studies.

The main reason for using N-of-1 trials in developing countries has been lack of evidence – that is “uncertainty due to lack of RCT evidence.” Five of the studies were done with the intention to test the efficacy of TCM. Quality use and tailoring of TCM to individual needs are crucial partly because many developing countries still rely on traditional medicine and partly because there is a significant lack of RCT evidence in this area. N-of-1 trials are indicated whenever there is lack of evidence regarding the comparative effectiveness of treatments being considered for an individual patient [40]. Johnston and Mills [17] specifically recommended the use of N-of-1 trials to make traditional and complementary medicine more widely available to appropriate patients without incurring undue public health consequences.

One of the trials [33] enrolled the majority [41] of participants. In this study no one responded to the placebo, but more than half, 28 (60%) did not benefit from the active treatment. Interestingly, all were willing to stop the medicine. After completing the trial, around two thirds (69%) of participants changed their medication in a direction consistent with the trial results. Only one patient was involved in the sixth [34] study. This patient was suffering from a dry cough secondary to interstitial pneumopathy and she had not responded to several treatments including antitussive agents. Following the N-of-1 trial of tramadol vs placebo, her cough and quality of life improved and the patient continued taking tramadol.

## Discussion

This review assessed the extent of use, purpose and treatment outcome of N-of-1 trials in developing countries. We concluded that the degree to which N-of-1 trials have been used was low. We identified and discussed three potential uses of N-of-1 trials to improve the standard of clinical care in resource-poor settings.

Only six studies were identified, with five from China (Table 2). In contrast, many developed countries (Australia, New Zealand, Canada, United States and countries in Europe) have been involved in a range of N-of-1 trials of modern medicines [23]. This difference is due to the high reliance on traditional medicine in developing countries, but with insufficient evidence of their effectiveness [42].

The overall completion rate was 93%, which is better than the figure reported in a previous review of N-of-1 trials in the medical literature (80%) [23]. Slightly higher than two thirds (69%) of participants changed their medication in a direction consistent with the trial results (Table 4), which is higher than the previous review which reported that 54% of participants made subsequent treatment decisions consistent with the results of the trial [23].

N-of-1 trials require that the intervention has a rapid onset and washout [16–18, 43]. A particular concern in these studies is the possibility of a “carry over” of treatment effect which can compromise the validity of the result due to a bias towards the null. Due to lack of pharmacokinetic data available on the TCM therapies, which are often mixtures of herbs, it is impossible to assess whether the studies included in this review [33, 35–37] were of appropriate period length and whether the washout periods were adequate. It is, therefore, impossible to assess the validity of their findings. To address this, Johnston and Mills [17] recommends initiation of these tests only after an initial trial of therapy to assess effectiveness, onset of action and probable washout time, so as to produce a credible trial design. Only one study conducted a symptom-based preliminary study [32] to determine the onset and washout characteristics of the therapy under investigation.

There are many opportunities to apply N-of-1 tests in resource-limited countries. First, physicians can use them as a clinical care tool to provide optimal therapy for individual patients. For example, researchers in Brazil were able to find an optimal therapy (tramadol) for a patient who had been suffering from dry cough who was not responsive to several antitussive drugs [34] (Table 2). One of the problems in clinical care is heterogeneity of treatment effects among individual patients [41, 44–49]. As the majority of clinical trials are carried out in Caucasian populations and take little account of factors that affect response to a medicine (other populations’ genetics, environments and lifestyles), there could be a higher risk in applying results of these trials directly to the medical care of patients in developing countries. Therefore, if clinically appropriate, N-of-1 trials could play a significant role in promoting safe, individualized medicine.

Additionally, Traditional Herbal Medicine (THM) use is common in developing countries, use ranging from 40% of people in China to 80% of people in Africa [42]. Though the contribution of traditional medicines to public health in developing countries is significant, evidence from RCTs or other controlled trials is either insufficient or lacking in most cases. In this review, five of the studies included [32, 33, 35–37] have used N-of-1 trials in THM (Table 2).

Second, N-of-1 trials can contribute to quality assurance of medicines in developing countries. These countries lack adequate capacity to control the quality, safety and efficacy of the medicines circulating in their market [50]. Some of these countries do not require proof of bioequivalence to ensure quality of generic drugs. For example, a 2014 report stated that drugs exported from India to Africa were of poorer quality than those sent elsewhere [14]. The application of this tool by health care professionals could be useful in recognizing clinically inferior drugs and thus contribute to the identification of sub-standard products [26]. Currently, a pilot N-of-1 trial is underway in Ethiopia to test the feasibility of these studies to generate therapeutic equivalence data on generic drugs that do not have proof of bioequivalence.

Third, aggregating multiple N-of-1 trials [18, 51] is useful to address lack of evidence on therapy. In this review, two of the trials conducted in China [33, 35] reported a population treatment effect by meta-analysis.

Fourth, N-of-1 trials can be used to identify cost-effective medications. Often, chronic diseases require lifelong treatment, but there is limited capacity for people in developing countries to afford even essential medicines. Beyond their potential for promoting patient-centered care, N-of-1 trials may have additional pragmatic value in identifying affordable treatment options [21, 22, 26]. Compared to drugs made in developing countries (both locally made and imported from other developing countries), drugs imported from developed countries are highly expensive. High drug expenses for those of limited resources may mean a choice between medicines and necessities such as food or clothing [52]. By objectively evaluating the effectiveness of drugs made in developing countries [53], N-of-1 trials can help physicians choose the cheapest of the effective drugs available.

To address the clinical inconvenience factor from the additional trial periods and subsequent length of N-of-1 trials compared to the standard trial of therapy, a major barrier for their widespread adoption, researchers have suggested the use of a novel N-of-1 trial designs such as a mixed-methodology add-on N-of-1 trial [54]. This involves conducting N-of-1 trials among apparent responders from a parent traditional RCT in research settings. This design addresses many of the concerns with both conventional RCTs and N-of-1 trials. Has different uses in complementary and alternative medicine research [54]. Also, the academic literature that guides design, analysis and reporting of N-of-1 trials [26, 27, 31, 55, 56] are widely available. The development of this groundwork can guide the broader applications of N-of-1 trials in resource-poor settings, becoming more important with the increasing focus on individualized medicine.

However, there are considerable operational and strategic barriers to consider in developing them:

- Logistic (a well-equipped research facility, placebo, etc.) and operational (administrative and patient recruitment) challenges
- Regulatory issues which are complicated by the lack of laws on emerging clinical trial methods such as N-of-1 trials
- Most physicians in developing countries have limited access to, and knowledge of, interpreting the results of RCTs, which would also apply to N-of-1 trials; physicians in resource-poor settings may have difficulties in obtaining information about N-of-1 trials and may have little knowledge of the added-value that N-of-1 trials can provide
- Moreover, the barriers already documented to conducting clinical trials in developing countries [37, 57, 58] and the specific barriers reported for implementing N-of-1 trials in developed countries (physicians’ time, physicians’ acceptance, drug companies’ acceptance, patient willingness, and cost [59–61]) may challenge the wider use of N-of-1 trials in this setting

### Limitations

This review has some limitations. Even though we included many databases, language was a major barrier in searching local databases. This may have excluded potentially useful articles from developing countries. Most of the conclusions of this review are derived from only six articles with potentially a high risk of bias in most of them.

### Future directions

The key implication of the sparse academic literature included in this review is that N-of-1 trials, designed and conducted well, could be possible in developing countries.

Physicians in developing countries may be able to use these trials to optimize clinical care for individual patients, while at the same time contributing to quality assurance.

Below are some strategies that can address operational and strategic barriers:

- Development of local initiatives on patient-centered research, along with international and local partnership for capacity building and funding, is needed
- Collaboration and resource-sharing to establish and standardize regulatory structures that appreciates the various spectrum of emerging research designs
- Education/training of health professionals would be required

## Conclusion

This paper reviewed the use and scope of N-of-1 trials in resource-poor settings and highlighted the potential roles of N-of-1 trials in clinical care in developing countries. In the context of the increasing trend towards PM and concerns about the quality of drugs in developing countries, N-of-1 trials may be feasible tools to introduce patient-centered medicine and improve the quality of medicines in developing countries, if the substantial barriers can be addressed.

### Additional file

Additional file 1: PRISMA 2009 Checklist. (DOC 62 kb)

## Appendix

**Table 5:** Detailed assessment of study quality

Author (date)	Sources of risk of bias							
	Selection bias (random sequence generation)	Selection bias (allocation concealment)	Reporting bias (incomplete outcome data)	Reporting bias (selective reporting)	Performance bias (blinding of participants and clinicians)	Inadequate cycles (risk of error – especially type 2)	Appropriateness of treatment for design	Appropriate washout period
Huang, et al., (2014) [32]	*Low* Method of random sequence generation is describe.	*Low* Independent pharmacist assigned treatments	*Low* All outcome data are reported	*Low* All outcome data are reported	*Low* Method of blinding is adequately described	*Low* 3 cycles were conducted	*Unclear* Biochemical and pharmacokinetic information of the TM is not known	*Low risk* Adequate washout period based on preliminary study
Yuhong, et al., (2012) [33]	*Low* Method of random sequence generation is described	*Low* Independent pharmacist assigned treatments	Low Acceptable reasons for missing data are given	*Low* All outcome data are reported	Unclear Method of blinding is not adequately described	Low 3 cycles were conducted	Unclear Biochemical and pharmacokinetic information of the TM is not known	High 2 days of washout period decided speculatively
Wang et al., (2010) [35]	*Low* Method of random sequence generation is described	*Low* Independent pharmacist assigned treatments	Low Acceptable reasons for missing data are given	*Low* All outcome data are reported	*Low* Method of blinding is adequately described	Low 3 cycles were conducted	Unclear Biochemical and pharmacokinetic information of the TM is not known	High The length of washout period is not well justified
Yu, et al., (2012) [36]	*Low* Method of random sequence generation is described	Unclear The independence of the person who assigned treatments is not well described	Low All outcome data are reported	Low All outcome data are reported	High Method of blinding is not adequately described	Low 3 cycles were conducted	Unclear Biochemical and pharmacokinetic information of the TM is not known	High The length of washout period is not well justified
Zhang, et al., (2012) [37]	*Low* Method of random sequence generation is described	Unclear The independence of the person who assigned treatments is not well described	Low All outcome data are reported	Low All outcome data are reported	High Method of blinding is not adequately described	Low 3 cycles were conducted	Unclear Biochemical and pharmacokinetic information of the TM is not known	High The length of washout period is not well justified
Louly, et al., (2009) [34]	Low Method of random sequence generation is described	Low Treatment assigned by a researcher who had no contact with the patient or result	Low All outcome data are reported	Low All outcome data are reported	Low Method of blinding is adequately described	Low 3 cycles were conducted	Low The design is suitable for tramadol	Low Adequate washout period

*TM* trial medication

## Abbreviations

CENT: CONSORT extension for reporting N-of-1 trials
EBM: Evidence-based medicine
FDA: Food and Drug Administration
GNI: Gross National Income
HN: Hypertension
PM: Personalized medicine
RCT: Randomized controlled trial
US: United States
WHO: World Health Organization
